# Effects of Different Methods of Isolation on Volatile Composition of* Artemisia annua* L.

**DOI:** 10.1155/2018/9604183

**Published:** 2018-08-19

**Authors:** Danijela Vidic, Amira Čopra-Janićijević, Mladen Miloš, Milka Maksimović

**Affiliations:** ^1^Department of Chemistry, Faculty of Science, University of Sarajevo, Sarajevo 71 000, Bosnia and Herzegovina; ^2^Faculty of Chemistry and Technology, University of Split, 21000 Split, Croatia

## Abstract

In order to determine influence of extraction method on volatile oil composition of* Artemisia annua* L., steam distillation, hydrodistillation, organic solvent extraction, and headspace sampling have been applied. The relative abundance of volatile compounds from the odorous aerial parts of* A. annua*, obtained by different extraction techniques, was analyzed by GC-MS. Exactly fifty constituents were identified. The leaf and flower essential oil yield ranged from 0.9 to 2.3% (v/w). Oxygenated monoterpenes were predominant in all samples ranged from 42.6% for steam-distilled fraction of petroleum ether extract to 70.6% for headspace of plant material. Essential oils isolated by steam distillation and hydrodistillation indicate that* A. annua* belongs to artemisia ketone chemotype with its relative content of 30.2% and 28.3%, respectively. The principal constituent in headspace sample of plant material was also artemisia ketone (46.4%), while headspace of petroleum ether extract had camphene (25.6%) as the major compound. The results prove the combined approaches to be powerful for the analysis of complex herbal samples.

## 1. Introduction

Within the tribe Anthemideae of the family Asteraceae, with its nearly 100 genera,* Artemisia *is by far the largest and most distributed genus, comprising some 400-500 species including a number of which are important for their medicinal properties [1, 2].

The most famous among them is* Artemisia annua* L., sweet wormwood, a highly aromatic herb of Asian and Eastern European origin that is nowadays naturalized worldwide. This annual herbaceous plant with a strong fragrance is known as the only source of the traditional Chinese herbal medicine Qing Hao, which has been used for over 2000 years to alleviate fevers and to stop malarial attack. These ethnopharmacological applications led to the isolation of the sesquiterpene lactone endoperoxide artemisinin present in the aerial parts of the plant [3, 4].

This compound of a cadinene skeleton with a unique 1,2,4-trioxane segment, responsible for antimalarial activity of* A. annua*, was discovered in 1972 by Professor Tu's team. This discovery was recognized by her receipt of the Nobel Prize in medicine in 2015 [5, 6]. Due to this discovery, as one of the most important achievements for the natural products chemistry in last decades of the twentieth century*, A. annua* is now rated as one of the top ten industrial crops of the modern world.

Nowadays, artemisinin and semisynthetic derivatives of similar structure are being developed as a novel class of peroxide-based antimalarial drugs, with activity against multidrug-resistant strains of* Plasmodium falciparum* and cerebral malaria [7, 8]. Furthermore, the use of artemisinin-based combination therapy (ACT) is widely recommended by World Health Organization (WHO) in different countries as the most effective one against the drug-resistant parasites. Some data shows that artemisinin becomes cytotoxic in the presence of ferrous iron [9].

Following the discovery of the important anti-mullerian sesquiterpene lactone artemisinin from* A. annua* L., there have been extensive phytochemical investigations of this species resulting in the description of many compounds such as terpenoids and flavonoids, as the most characteristic secondary metabolites. More than 150 natural products were reported to belong to different chemical structure types [10].

*Artemisia annua* is also valued for its essential oil of which the characteristic sweet aroma has been described as grassy, fresh, and bitter with a camphoraceous nuance. The commercial significance of the essential oil is limited, but it is sometimes used in fragrances, in perfumery, and cosmetic products. The essential oil has been reported to possess antimicrobial [11], antioxidant [12, 13], leishmanicidal [14], and cytotoxic [15] activities.

The essential oil composition of this medicinal plant, which exhibits a chemical variability, has been studied thoroughly and hundreds of components have been identified up to date [16]. The great diversity was found in the essential oil components of* A. annua* with monoterpenes being principal constituents [10] that contribute to the characteristic fragrance of this medicinal species. The monoterpene fraction of the essential oil from* A. annua *is composed of a diverse array of structures from the prosaic, regular monoterpenes such as *α*-pinene, 1,8-cineole, and camphor, to the rearranged, such as fenchol and camphene hydrate, and to the irregular, such as artemisia alcohol, artemisia ketone, and santolina triene which give the plant a sweet scent. Depending on the variety, dominant compounds were artemisia ketone and camphor [17–20], camphor and 1,8-cineole [21–23], *α*-pinene and pinocarvone [24], and artemisia ketone and 1,8-cineole [17, 25]. The exception is a chemotype with phenolic compounds thymol and carvacrol as the principal constituents [26], and varieties with no artemisia ketone detected [27], and with oxygenated sesquiterpenes as the major constituents [28].

The present work is a continuation of our study on chemical composition of essential oil from* A. annua* of Bosnian origin [20, 28]. Traditionally, this fragrant plant is used for crafting of aromatic wreaths, flavouring of spirits and insect repellent.

Previously we demonstrated that* A. annua *cultivated near Sarajevo, Bosnia, belongs to artemisia ketone-camphor chemotype and its essential oil shows antioxidant and antimicrobial activity [20].

In the present work, comparison of the volatile compounds from the leaves and flowers of the wild grown* A. annua*, obtained by different methods of isolation, steam distillation, hydrodistillation, organic solvent extraction, and headspace technique, was investigated.

## 2. Experimental

### 2.1. Plant Material

The aerial plant parts of* Artemisia annua* L. were collected from natural habitat in central part of Bosnia. Investigated plant was harvested in the full flowering stage and put to dry in ambient condition. A voucher specimen was deposited at the Faculty of Science, University of Sarajevo.

### 2.2. Chemicals and Reagents

All reagents were of the highest purity available and obtained from the Sigma-Aldrich Chemical Company (Germany).

## 3. Materials and Methods

### 3.1. Sample Preparation

Air-dried plant material was subjected to standard steam distillation (Sample B) and hydrodistillation (Sample C) (50 g for each), during 2 h. The essential oils were extracted with dichloromethane and dried over anhydrous sodium sulphate.

Plant material was also extracted by Soxhlet extraction (3 h) with petroleum ether as a solvent. Solvent was removed using rotary evaporator (temperature of water bath was 30°C) and crude extract was subjected to the standard steam distillation (Sample E), in order to isolate volatile components of the extract.

Floral and leaf aroma, of dry plant material (10 g) (Sample A), as well as scent of petroleum ether extract (Sample D), were collected using dynamic headspace sorption on Dräger charcoal tubes #6728631 with sorption agent coconut shell charcoal; trapping time was 3 h, at ambient temperature. Trapped volatiles were eluted with dichloromethane.

### 3.2. GC-MS Analysis

The analysis of volatile compounds was performed using two GC-MS instruments and two columns of different polarity. First GC-MS analysis was carried out on the Perkin Elmer Mass, fitted with a fused-silica PE-5 (5% phenyl methyl siloxane) capillary column (30 m x 0.25 mm, 1 *μ*m film thickness), and coupled to a Turbo Mass Auto System XL. Column temperature was programmed from 55°C at 10°C/min to 140°C held 1 min isothermal and then 10°C/min to 245°C. Helium was used as carrier gas (1.1 mL/min). Other operating conditions were as follows: injector temperature, 240°C; detector temperature, 280°C; injection volume, 1 *μ*L. Ionization of the sample components was performed in the EI mode, (70 eV), with scan range 20-555 amu, and scan time 1.60 s.

The GC-MS analysis on the polar column was carried out using Hewlett Packard GC-MS system (GC 5890 series II; MSD 5971A, Hewlett Packard, Vienna, Austria). The fused-silica HP-20 M polyethylene glycol column (50 m x 0.2 mm, 0.2 *μ*m thickness) was directly coupled to the mass spectrometer. The carrier gas was helium (1 ml/min). The program used was 4 min isothermal at 70°C, then was 4°C/min to 180°C, and was held 10 min isothermal. The injection port temperature was 250°C, and the detector temperature was 280°C. Ionization of the sample components was performed in the EI mode (70 eV). The linear retention indices for all compounds were determined by injection of the sample with a solution containing the homologous series of C_8_-C_24_* n*-alkanes under the above conditions in order to calculate the retention indices using generalized equation [29].

The identification of the essential oil constituents was accomplished by comparing retention indices from the literature data [30] and mass spectra by computer library search (HP Chemstation computer library NBS75K.L, NIST/EPA/NIH Mass Spectral Library 2.0 and Mass Finder 4 Computer Software and Terpenoids Library).

## 4. Results and Discussion

Chemical characterization of volatile profile of* A. annua*, using four different methods of isolation: headspace, steam distillation, hydrodistillation, and solvent extraction has been done.

Hydrodistillation (HD) was applied as one of the simplest conventional ways to separate the essential oil from different parts of the plants. Steam distillation (SD) used in this study is similar and standard method for essential oils isolation. However, the main restrictions of HD as well as of SD are as long extraction times, the possibility of the decomposition, and/or loss of heat-sensitive compounds during the extraction. In contrast, headspace method is a very facile, sensitive, and solvent-free sampling and concentration technique for the determination of volatile compounds without destroying plant material. In addition, petroleum ether was chosen for isolation of various compounds from plant material soluble in nonpolar solvent which is easy to remove after extraction in Soxhlet apparatus.

The yield of clear yellowish coloured hydro-distilled essential oil, sample B, and steam-distilled essential oil, sample C was 1.0% and 0.9%, respectively, while sample E, obtained by extraction with petroleum ether as a solvent, followed by steam distillation, was recovered in yield of 2.3%. The yield is expressed as % of extracted essential oil relative to the mass of dry plant material.

Chemical composition of three distilled essential oils and two headspace samples was determined by detailed gas chromatography-mass spectrometry GC-MS analysis. The identification of the plant fragrance and other extractable plant components was performed using two GC columns, polar HP-20 and semipolar PE-5. Distribution of the volatile constituents, their RI indices, and relative amounts are presented in Table 1.

Exactly 50 components were identified in all five samples, many of which occurred only in amounts below 1%. Three components, ethyl 2-methyl butanoate, santolina triene,* cis*-3-hexenyl 2-methylbutanoate, were found only in sample A, isolated by headspace technique of dry plant material. Seven compounds were found in all samples: *β* -myrcene, 1,8-cineole, artemisia ketone, artemisia alcohol, camphor, *β*-caryophyllene, and (*E*)-*β* -farnesene, and among them the most abundant were irregular monoterpene artemisia ketone (16.5%-46.4%) and regular monoterpene camphor (7.5%-24.0%).

The highest number of total identified constituents was 47 in the sample B, representing 96.1% of the essential oil. The lowest number of identified components (11) was in headspace sample D comprising 97.8% of the total components detected. When grouping the components into different terpenoid types, there were found eight monoterpenes hydrocarbons (MH), 17 oxygenated monoterpenes (MO), 11 sesquiterpene hydrocarbons (SH), five oxygenated sesquiterpenes (SO), and one oxygenated diterpenes (DO). Most abundant compounds for all samples were MO, ranged from 42.6% (sample E) to 70.6% (sample A).

All heat-involving isolation procedures have been shown to differentiate the volatile fraction profile as analyzed by GC-MS. Although essential oils obtained by steam distillation and hydrodistillation showed prominent similarity in chemical composition, they still differ in quantitative composition, especially in their relative contents of the main constituents like camphor: 24.0% in sample B and 16.9% in sample C, respectively. Data on the essential oil composition of sample E, obtained by extraction with organic solvent followed by steam distillation, indicated the percentage concentration drop in terms of characteristic markers: artemisia ketone (16.5%) and camphor (20.0%) or absence of some compounds in the petroleum ether extract. The observed fact may be attributed to the effect of the heat applied; i.e., some of the compounds were degraded during distillation process or the concentration of compounds in the volatile fraction of the petroleum ether extract may be below the detection limit due to their loss during evaporation of the solvent.

Oxygenated sesquiterpenes (SO) were identified in all samples obtained by distillation process with caryophyllene oxide as the most abundant representative of this class (8.2% in sample E), while these compounds were not detected in the headspace samples.

Headspace of plant material (A) and essential oil (B i C) were of artemisia ketone chemotype. In headspace sample of petroleum ether extract (D), the main component was camphene (25.6%), while, in sample obtained by steam distillation of petroleum ether extract (E), the main component was camphor (20.0%) and camphene was not detected. Four high volatile compounds, *α*-pinene, camphene, sabinene, and *β*-pinene, comprising 43.7% of sample D, were not found in sample E where steam distillation was applied.

Comparing the emission from excised leaves and flowers and from crude petroleum extract of plant material, it can be concluded that number of components responsible for scent of plant material (sample A) are more numerous than volatiles emitted from petroleum ether extract (sample D). All 11 components identified in sample D were also present in sample A. Sample A is almost twice more heterogeneous than sample D, where 20 components comprised 96.5% of total identified constituents.

Literature survey revealed a few articles on headspace sampling of* A. annua* aroma performed by solid‐phase microextraction (SPME). Comparing our results on the plant volatile profile with previously published data, there are great differences in both qualitative and quantitative composition. The variability of chemical composition could be effected by many factors, such as genotype, geographic location, cultural practices, used parts of the plant, and mode of extraction of volatiles used, so the types of major volatile compounds in shoots differ from those detected in roots of cultivated* A. annua* [31]. Headspace SPME analysis of artemisinin-rich* A. annua* cultivars showed the absence of artemisia ketone [32] known as a characteristic volatile biomarker of wild-type species [33].

## 5. Conclusions

The data on volatile profile of* Artemisia annua *growing wild, presented in this study, confirm the already observed for the essential oil, the relative percentages, however being different due to the different extractions and analytical protocols. Significant differences were found for GC pattern of headspace samples from that prepared by distillation. Application of nondestructive headspace method for extraction of the volatiles is considered to prevent a damage of the thermal-sensitive molecules, thus providing a better approach of the compounds primarily responsible for the characteristic odour of dry herb. Apparently, the content and composition of volatiles are greatly influenced by technique of isolation. The use of headspace proves to be crucial in order to provide reliable insight into* Artemisia annua* chemistry.

## Figures and Tables

**Table 1:** Volatile constituents of *Artemisia annua* L. obtained by different methods of isolation.

**Compound**	**RI** ^**a**^	**RI** ^**b**^	**Sample (**%**)**				
			**A**	**B**	**C**	**D**	**E**
Ethyl 2-methyl butanoate	869		0.3	-	-	-	-
Santolina triene	910		0.5	-	-	-	-
Artemisia triene	929		-	0.4	0.2	-	-
*α*-Pinene	937	1043	0.3	t	-	11.9	-
Camphene	951	1061	6.5	0.8	0.6	**25.6**	-
Sabinene	972	1117	0.5	0.2	t	6.2	-
*β*-Pinene	976	1102	t	t	-	t	-
*β*-Myrcene	984	1145	**17.1**	3.7	4.2	1.7	0.9
Yomogi alcohol	997	1370	-	0.5	3.9	-	-
*p*-Cymene	1025	1244	-	t	t	-	-
1,8-Cineole	1031	1193	8.5	5.3	5.4	18.3	0.3
Artemisia ketone	1065	1330	**46.4**	**30.2**	**28.3**	**24.5**	**16.5**
*cis*-Sabinene hydrate	1076	1524	-	t	-	-	-
Artemisia alcohol	1087	1475	2.5	3.1	2.2	0.5	2.8
*trans*-Sabinene hydrate	1100	1423	-	0.8	0.4	-	0.7
*trans*-Pinocarveol	1141	1603	-	0.5	0.4	-	t
Camphor	1151	1476	**13.2**	**24.0**	**16.9**	**7.5**	**20.0**
Pinocarvone	1167		-	0.7	0.4	-	-
Borneol	1172	1657	-	t	t	-	-
Terpinen-4-ol	1175	1560	-	0.7	0.7	-	1.5
*p*-Cymen-8-ol	1181		-	t	t	-	t
*α*-Terpineol	1187	1650	-	1.3	0.7	-	0.8
Myrtenal	1209	1580	-	0.7	0.4	-	-
Myrtenol	1210	1730	-	1.0	0.4	-	-
*cis*-3-Hexenyl 2-methylbutanoate	1217		0.1	-	-	-	-
Hexyl 2-methylbutanoate	1219		-	0.6	0.4	-	0.5
3-Tetradecene	1274		0.2	0.2	0.2	-	-
*p*-Cymen-7-ol	1280		-	t	t	-	-
Piperitenone	1342	1856	-	1.1	0.8	-	-
Eugenol	1358	2091	-	0.9	1.2	-	-
*α*-Copaene	1384	1466	-	0.7	0.7	-	0.5
Phenyl methyl pentanoate	1387	1813	-	0.1	0.4	-	0.5
*β* -Cubebene	1390	1666	-	1.7	0.8	-	-
*β*-Elemene	1396		-	0.8	0.6	-	-
*β*-Caryophyllene	1426	1565	0.1	3.1	3.9	1.3	1.2
*α*-Humulene	1456	1620	t	t	0.6	-	-
(*E*)-*β*-Farnesene	1458	1634	0.2	4.2	4.1	0.4	t
*τ*-Selinene	1476	1646	0.1	1.8	1.3	-	-
*γ*-Muurolene	1478		0.1	t	t	-	-
Germacrene -D	1493	1689	-	0.6	0.6	-	-
*β*-Selinene	1495	1675	t	0.4	0.3	-	1.0
Viridiflorene	1497	1663	t	t	t	-	-
Isobornyl-3-methylbutanoate	1513		-	t	-	-	0.5
Caryophyllene oxide	1590	1930	-	2.4	2.6	-	**8.2**
1-*epi*-Cubenol	1619	2006	-	0.7	0.8	-	1.8
Selina-3,11-diene-6-*α*-ol	1631		-	0.8	0.6	-	2.7
Selin-11-en-4-*α*-ol	1649		-	0.5	0.4	-	1.9
*α*-Bisabolol	1664	2164	-	0.4	0.5	-	1.4
Hexahydrofarnesyl acetone	1779		-	0.4	2.4	-	0.7
Phytol	1970		-	0.6	-	-	-

Monoterpenes hydrocarbons (MH)			24.9	5.1	5.0	45.4	0.9
Oxygenated monoterpenes (MO)			70.6	69.9	60.9	50.8	42.6
Sesquiterpene hydrocarbons (SH)			0.5	13.3	13.0	1.7	2.6
Oxygenated sesquiterpenes (SO)			-	4.9	4.9	-	15.9
Oxygenated diterpenes (DO)			0.6	2.2	4.6	-	2.3
Others (O)			-	0.6	-	-	-

**Total **			**96.5**	**96.1**	**88.3**	**97.8**	**64.3**

RI^a^, retention indices relative to C_8_–C_24_, *n*-alkanes on the PE-5 capillary column; RI^b^, retention indices relative to C_8_–C_24_, *n*-alkanes HP-20M capillary column; sample **A**: headspace of plant material of *A. annua*; sample **B**: essential oil isolated by steam distillation; Sample **C**: essential oil isolated by hydrodistillation; sample **D**: headspace of petroleum ether extract; sample **E**: steam distillation of petroleum ether extract; %: relative percentage obtained from peak area; t<0.1%. Percentages of dominant compounds are given in bold.

## Data Availability

The data used to support the findings of this study are available from the corresponding author upon request.
